# Characterizing Inland Pacific Northwest American Viticultural Areas with Geospatial Data

**DOI:** 10.1371/journal.pone.0061994

**Published:** 2013-04-16

**Authors:** Ian-Huei Yau, Joan R. Davenport, Richard A. Rupp

**Affiliations:** 1 Crop and Soil Sciences, Washington State University, Prosser, Washington, United States of America; 2 Crop and Soil Sciences, Washington State University, Pullman, Washington, United States of America; DOE Pacific Northwest National Laboratory, United States of America

## Abstract

American Viticultural Areas are officially recognized appellations for wine grapes (*Vitis vinifera* L.). They represent not only geographic identification for growers, but also economic significance through price premiums for grapes from desirable appellations and wines sourcing grapes from such appellations. Petitions for establishment and official descriptions of American Viticultural Areas in the inland Pacific Northwest have traditionally relied on general descriptions of physical attributes and data from point measurements, namely weather stations. Examination of spatial datasets in a geographic information system provides a more holistic means of assessing viticultural areas and a spatially continuous representation of an area. Comparison of spatial datasets to official appellation descriptions largely corroborate petitioners' claims, often with greater detail, but also highlight some shortcomings of official appellation descriptions. By focusing on spatial data representing environmental factors most important to wine grape production, viticultural areas can be described more thoroughly and accurately and appellations may be more appropriately delineated. We examined inland Pacific Northwest American Viticultural Areas with a geographic information system approach, illustrating the utility of spatial datasets in characterization and delineation of American Viticultural Areas.

## Introduction

The inland Pacific Northwest (IPNW), the area east of the Cascade Mountains in Washington state, Oregon and Idaho, has developed into a world-class wine grape (*Vitis vinifera* L.) producing region. The vast majority of wine grape production occurs in Washington state, which currently grows nearly 18,000 ha of wine grape vineyards, a 395% increase over the last 18 years [1]. Washington state is second only to California in wine grape production in the United States [2].

**Figure 1:**
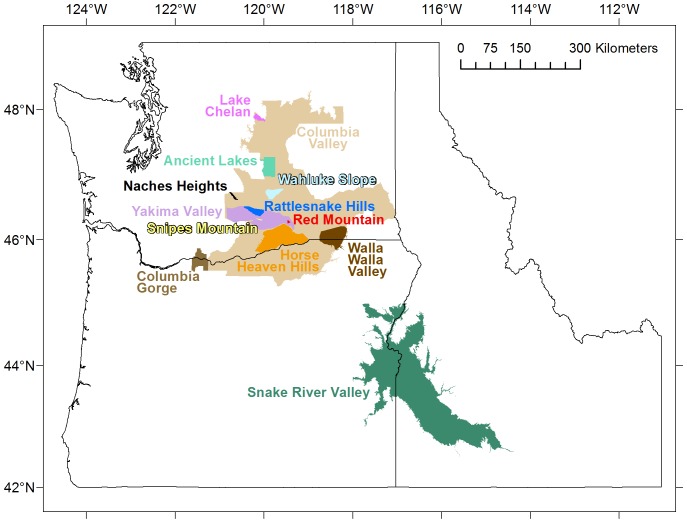
American Viticultural Areas of the inland Pacific Northwest.

The IPNW hosts thirteen American Viticultural Areas (AVAs) acknowledged by the Alcohol and Tobacco Tax and Trade Bureau (TTB) on the basis of national or local name recognition, usage and distinguishing features (e.g., climate, geology, soils, physical features and elevation). Three AVAs, Columbia Valley, Columbia Gorge and Walla Walla Valley, share area between Washington and Oregon. One more appellation, Snake River Valley AVA, straddles the border of Oregon and Idaho. These thirteen AVAs comprise the major grape growing regions of the IPNW (Fig. 1). Burgeoning viticulture in the Snake River Valley is receiving proactive research in the form of cultivar trials [3].

The concept of *terroir* is embedded within the nature of appellations despite the widely variable sites found in many AVAs, especially massive ones like Columbia Valley AVA. The distinction between what an actual vineyard bestows upon a grape and the perception of fruit characteristics from a given AVA often comes down to the physical features of a vineyard and its viticultural practices versus the marketing surrounding an AVA or the notable exploits of outstanding vineyards within the area. Another highly variable aspect of *terroir* is winemaking practice. Unlike Old World appellations, AVAs face fewer enological restrictions and AVA boundaries arguably reflect more physical characteristics of vineyard sites and less consistent anthropological influence. Wine prices were strongly determined by appellation designation, but not by specific site attributes in the Willamette Valley [4]. Delineation of AVAs thus represents a serious economic concern for grape growers and winemakers.

Traditional approaches to appellation delineation rely on point measurements of climatic attributes, a general sense of physical attributes, soil survey and topographic maps. Particularly with climatic data, this leaves much to be desired as mesoclimate (sub-regional to vineyard scale) can vary widely over relatively short distances [5]. Geomorphologic features may be described vaguely or broadly to encompass large areas. Contour lines on topographic maps are often used to delineate portions of appellation boundaries with inaccurate descriptions of maximum and minimum elevations within the proposed area.

Climate is perhaps the ultimate limiting factor in *V. vinifera* production. Growing degree-day (GDD) accumulation is one common method of measuring relative heat accumulation within the growing season. Growing degree-day accumulation for *V. vinifera* is calculated as the summation of average temperatures less a threshold of 10°C between 1 April and 31 October. Amerine and Winkler [6] developed five categories of general wine styles from *V. vinifera* grapes in California based on this index of heat accumulation.

Frost-free days (FFD), the period between the last spring and first autumn frosts (0°C), is frequently examined in determining the suitability of an area for grape production [7]. Frost-free days indicate growing season length and serve as a proxy of the period over which *V. vinifera* can develop and ripen. Precipitation during the growing season is linked to primary infection and subsequent favorable microclimate for grape powdery mildew (*Uncinula necator* (Schwein.) Burrill [syn. *Erysiphe necator* Schwein.]), the most common fungal disease of grapes in the IPNW [8]. High humidity accompanied by moderate temperature provides favorable conditions for powdery mildew development [9], [10].

Topographic suitability relates to the physical ability to manage a vineyard (i.e. ability for machinery to safely operate on a site) and influence over mesoclimatic (sub-regional to vineyard scale) conditions. Modern vineyard machinery readily operates on slopes of up to 15% if vine rows are parallel to the slope [11] and high-end, self-leveling equipment can handle side slopes of 30 to 35% [12]. Southern-facing slopes are often given greatest preference in the Northern Hemisphere due to higher levels of solar insolation, and consequently heat accumulation. Moderate slopes (5 to 15%) are considered the best sites for grape production [13]. Katabatic cold air flow and subsequent lower ambient temperatures can be severely limiting to shoot development, inflorescence development, pollen formation and flower fertilization. Sloped sites can reduce cold air pooling as they promote air drainage to alternate locations. Sites located above potential cold air pools may also benefit from additional elevation through lower daytime temperatures which can promote fruit quality in hot regions [14]. Unfortunately, slope alone cannot predict mesoclimate conditions and sites must be considered within the greater context of surrounding topography, obstructions to air flow and prevailing winds [15]. Ideal elevations are region-specific and vary with geography and macroclimate. Relative elevations (i.e. height above a valley floor) are more important than absolute elevation above mean sea level [14], [16].

*V. vinifera* tolerates a wide range of soil conditions and no one soil type can be proven superior. Nutrient availability and moisture management are the two major areas of importance governed by soil characteristics [13]. The IPNW hosts a predominance of vineyard sites on Quaternary sediments overlying Miocene basalt. These are commonly Aridisols, the order of soils formed under arid or semi-arid climates, with upper horizons composed of loess (aeolian silt) or sand sheets and lower horizons consisting of stratified silty to gravelly flood sediments [17].

Soil drainage is a critical factor in grape production [13], [18]. Waterlogged soils retard vine growth, hinder mechanical operations in the vineyard and favor the development of several root diseases and chlorosis in calcareous soils [19]. Free-draining soils maintain oxygen concentrations near roots and facilitate moderate water stress with proper irrigation management [20].

Unrestricted soil drainage to a depth >2 to 3 m is recommended for vineyards in most situations [18], [19]. Failla et al. [21] found grapevine roots at depths >3 m during soil surveys and vines may grow roots to depths >30 m if no impenetrable barriers are present [20]. Only under severe water stress will *V.* vinifera access substantial water from >2 m. Vines draw most of their nourishment from a depth of 50 to 100 cm and readily transpire water from depths up to 2 m, but root concentrations may shift to greater depths under conditions such as competition with vineyard floor cover crops [20], [22]. Shallow soils where root penetration is problematic are considered unsuitable for grape production and increase the likelihood of waterlogging [19]. Good soil drainage, along with greater depth, increases grapevines' incentive to grow robust, perennial root structures. Roots act as storage organs for carbohydrates, water and other nutrients, which are important for initial growth of shoots and roots when vines break dormancy [14], [20], [23].

Under ubiquitous irrigation, IPNW vineyards offer the most flexibility to consistently ripen quality crops when the available water-holding capacity (AWC) of soils is relatively low to moderate. Moisture needs of grapevines vary throughout the growing season and the ability of soils to maintain plant available levels of water without constant irrigation, as well as induce some level of water stress within relatively short time frames, is desirable [18], [24].

A readily measured soil characteristic determinant of potential nutrient availability is pH [13], [19]. Absorption of many nutrients is optimal in *V. vinifera* at approximately neutral to slightly acidic soil pH (6.6 to 7.2) [25]. Overly alkaline soils lead to deficiencies of phosphorus, iron, manganese, boron and zinc [18]. Acidic soils can generate toxic levels of aluminum, copper and manganese, induce phosphorus deficiency, restrict root growth and lead to grapevine nutrient and soil microbial imbalances [18], [26].

Using available spatial datasets and creating datasets from available data to assess AVAs provides a spatially continuous, more accurate means of describing an appellation. By examining IPNW AVAs with a geographic information system (GIS) approach, we will illustrate the utility of spatial datasets in characterization and delineation of AVAs.

## Materials and Methods

Geographic information system software packages used were ArcGIS 9.3.1 and 10×(Esri, Redlands, CA). Thematic maps were created from Soil Survey Geographic (SSURGO) databases using Soil Data Viewer 5.2 (NRCS, Lincoln, NE). Tabular data were spatially represented using geographic coordinates provided by responsible organizations. Spatial data were projected to UTM Zone 11 North, North American Datum, 1983 using bilinear sampling for continuous data where necessary. AVA boundaries were mapped following final rule descriptions in the Federal Register, the corresponding, georeferenced U.S. Geological Survey topographic quadrangle maps and topographic lines extracted from the National Elevation Dataset (NED) 1/3 arc-second digital elevation model (DEM) [27], [28].

Slope, solar insolation and aspect were calculated from the 1/3 arc-second NED DEM. Slope calculations were made in units of percent rise. Aspect calculations were made in degree units; areas with zero slope were classified as flat.

Solar insolation calculations were performed using the mean latitude of each DEM to 10^−12^ decimal degrees, a sky size of 40,000 cells, 14-day and two-hour intervals. Calculation guidelines recommend the extent of input DEMs be less than one degree latitude [29], but calculations of adjacent surfaces with combined extents of much less than one degree latitude showed notable differences in continuity. Most DEMs masked by county were too large for a single calculation of solar insolation. Digital elevation models were divided vertically whenever possible to maintain relatively constant mean latitude for each county (±0.0001°). Vertical divisions were made with overlap to account for effects of adjacent topography and improved continuity. Divided surfaces were later mosaicked using a blend of overlapping areas. Calculations were made from ordinal day 91 to 304 (1 April to 31 October). Thirty-two azimuth directions were used to calculate viewshed. Eight zenith and azimuth divisions were used to calculate the sky map. A uniform sky diffuse radiation model was used. A diffuse proportion of 0.3 and transmissivity of 0.5 were used, presumptive of generally clear sky conditions [29], [30]. Solar insolation surfaces do not represent actual solar accumulation over a climatically relevant period of record and are intended to represent sites' topographic exposure. Output surface units are Watt hours per square meter (Wh/m2).

Thematic maps of soil characteristics were created for drainage class, depth to any restrictive layer, available water-holding capacity (0 to 50 cm) and pH (0 to 50 cm). Dominant component or condition was used as the aggregation method. Vector data was converted to raster data with a resolution of 10 m to adequately represent soil map unit boundaries and match DEM resolution.

Heat accumulation was calculated using Parameter-elevation Regressions on Independent Slopes Model (PRISM) monthly normals, 1971–2000 [31]. This model produces official climate surfaces used by the U.S. Department of Agriculture. Monthly normal maximum and minimum temperatures were averaged for April through October. From these monthly average temperatures, a base of 10°C was subtracted, each surface was multiplied by days in the month and the resulting surfaces were summed. Additionally, a biologically effective degree-day (BEDD) surface was calculated limiting daily heat accumulation for each month to 9C° after Gladstones who observed phenological development in *V. vinifera* to be most active from 10°C to 19°C [18]. A surface of median FFD from 1971–2000 was calculated by subtracting PRISM surfaces of median last spring frost date from median first fall frost date. Monthly PRISM precipitation normals from 1971–2000 were summed for April through October. This growing season precipitation serves as a proxy for *U. necator* risk.

Principal components analysis (PCA) was performed for each IPNW AVA using the ArcGIS Spatial Analyst. Surfaces included in PCA were DEM, soil AWC, soil pH, depth to restrictive layer, GDD, FFD and growing season precipitation. Slope, aspect and solar insolation were derived from the DEM, so including these surfaces in PCA would introduce redundancy. Soil drainage is classified into qualitative categories and was omitted from PCA. Growing degree-day and BEDD surfaces were both derived from the same PRISM datasets and GDD surfaces were included in PCA because the limitation on heat accumulation in BEDD reduced observable variability. All datasets included in PCA were standardized to z-scores by subtracting the mean and dividing by the standard deviation [32].

## Results and Discussion

### Geospatial analysis of American Viticultural Areas

**Figure 2:**
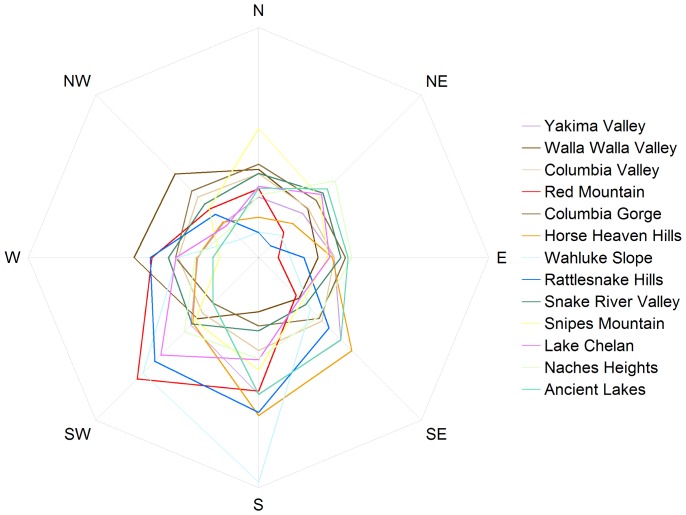
Aspect distribution by American Viticultural Area.

Table 1 shows means of site characteristics examined by AVA. Table 2 summarizes soil drainage class, a qualitative characteristic, with proportional representation of each class within each AVA. Figure 2 shows proportional distribution of aspect by AVA. These summaries do not represent realistically plantable area within each appellation, but merely the entire area within boundaries excluding water features from the National Hydrography Dataset. The availability of water for irrigation and the associated water rights are often a greater limitation than other facets of site suitability [17], [33]. Table 3 shows planted area of the three most common red and white *V. vinifera* varieties and other notable varieties for each available AVA based on a 2011 U.S. Department of Agriculture survey [1]. Discussion of AVAs proceeds in chronological order of establishment.

**Table 1:** Mean site characteristics by inland Pacific Northwest American Viticultural Areas sorted by chronological establishment.

	Topographic parameters			Soil parameters			Climate parameters			
	Elevation (m)	Slope (%)	Solar insolation (kWh/m2)	AWC (cm/cm)	Depth (cm)	pH	GDD (C°)	BEDD (C°)	FFD	PPT (mm)
Yakima Valley	331	5.2	1031.2	0.169	165	7.62	1371	1204	162	89
Walla Walla Valley	315	10.6	1007.2	0.186	190	7.36	1487	1269	200	197
Columbia Valley	462	11.3	1022.1	0.163	141	7.28	1278	1110	163	125
Red Mountain	227	7.7	1016.7	0.161	186	7.40	1501	1341	176	89
Columbia Gorge	405	22.8	983.5	0.163	158	6.48	927	908	150	200
Horse Heaven Hills	323	6.6	1031.7	0.169	179	7.52	1435	1236	174	98
Wahluke Slope	251	3.0	1027.9	0.120	174	7.73	1512	1281	173	73
Rattlesnake Hills	433	11.5	1031.6	0.176	108	7.67	1214	1119	148	105
Snake River Valley	874	10.8	1111.9	0.153	120	7.59	1339	1135	139	136
Snipes Mountain	279	17.4	994.4	0.189	166	7.63	1447	1284	167	80
Lake Chelan	488	24.5	951.6	0.143	170	7.43	1198	1055	168	112
Naches Heights	542	10.6	1026.2	0.169	142	7.10	1061	937	119	125
Ancient Lakes	386	4.3	1026.3	0.143	157	7.55	1380	1210	167	83

Abbreviations: AWC (available water-holding capacity), GDD (growing degree-days), BEDD (biologically effective degree-days), FFD (frost-free days), PPT (growing season precipitation).

**Table 2:** Summary of drainage classes by American Viticultural Area from Soil Survey Geographic database, masked to exclude water features, showing a predominance of soils classed as well drained.

	ED	SED	WD	MWD	SPD	PD	VPD
Yakima Valley	1.9%	5.6%	78.4%	0.6%	13.2%	0.3%	-
Walla Walla Valley	-	3.9%	86.8%	4.9%	4.2%	0.2%	-
Columbia Valley	6.6%	6.9%	83.0%	0.9%	2.0%	0.5%	0.1%
Red Mountain	0.6%	14.9%	84.5%	-	-	-	-
Columbia Gorge	-	0.2%	96.7%	-	2.9%	0.3%	-
Horse Heaven Hills	9.6%	9.4%	80.6%	-	0.3%	0.1%	-
Wahluke Slope	34.7%	33.7%	31.5%	-	<0.01%	-	-
Rattlesnake Hills	-	<0.01%	99.4%	-	0.6%	-	-
Snake River Valley	1.8%	4.6%	86.7%	0.8%	4.7%	1.2%	0.1%
Snipes Mountain	-	0.3%	99.2%	-	0.4%	-	-
Lake Chelan	-	0.9%	90.0%	9.1%	-	-	-
Naches Heights	-	<0.01%	98.3%	-	1.7%	-	-
Ancient Lakes	5.0%	27.5%	67.2%	0.1%	-	0.2%	-
Average	8.6%	8.3%	83.3%	2.8%	3.0%	0.4%	0.1%

Abbreviations: ED (excessively drained), SED (somewhat excessively drained), WD (well drained), MWD (moderately well drained), SPD (somewhat poorly drained), PD (poorly drained), VPD (very poorly drained).

**Table 3:** Planted area (ha) of three most common red and white *Vitis vinifera* varieties and other notable varieties in Washington American Viticultural Areas (AVAs).

	Red varieties					White varieties				
	Cabernet Sauvignon	Merlot	Syrah	Other	Total red	Chardonnay	Riesling	Pinot Gris	Other	Total white
Yakima Valley	679	930	295	80 Cabernet Franc, 32 Pinot Noir	2178	1278	1157	346	170 Sauvignon Blanc, 157 Gewürztraminer	3266
Walla Walla Valley	212	81	67	31 Cabernet Franc, 13 Petit Verdot	433	11	-	-	8 Viognier, 8 Sémillon	95
Columbia Valley	708	586	258	70 Cabernet Franc, 24 Malbec	1745	539	461	100	69 Sauvignon Blanc, 39 Gewürztraminer	1278
Red Mountain	272	74	70	17 Cabernet Franc, 8 Mourvèdre	478	-	-	-	8 Sémillon, 7 Sauvignon Blanc	37
Columbia Gorge	-	8	-	17 Pinot Noir, 2 Tempranillo	58	33	9	28	30 Gewürztraminer	102
Horse Heaven Hills	1344	828	306	107 Cabernet Franc, 51 Grenache	2824	717	354	56	199 Sauvignon Blanc, 36 Viognier	1459
Wahluke Slope	769	623	173	56 Cabernet Franc, 43 Malbec	1792	398	344	-	49 Gewürztraminer, 17 Sauvignon Blanc	898
Rattlesnake Hills	118	149	31	26 Cabernet Franc, 8 Petit Verdot	361	48	183	19	11 Viognier, 3 Chenin Blanc	286
Snipes Mountain	57	45	32	-	154	60	28	-	-	132
Lake Chelan	3	-	15	17 Pinot Noir, 2 Sangiovese	51	5	17	5	11 Gewürztraminer, 6 Viognier	49

Data for Snake River Valley, Naches Heights and Ancient Lakes AVAs were unavailable.

Adapted from [1].

The approximately 2,900 km^2^ Yakima Valley AVA became the first appellation in Washington state on 4 May 1983. The Federal Register describes the western portion of the AVA as a vast, flat expanse and the eastern portion as gently sloping north of the Yakima River [34]. It is the second flattest of IPNW appellations. Spanning from 46°08'N to 46°31'N latitude, the area registers the fourth highest solar exposure.

When the petition for establishment of Yakima Valley AVA was filed, it was stated by the petitioner that most vineyards were planted on two soil associations, Warden-Shano and Scootenay-Starbuck silt loams with varying depths over basalt and/or gravel [34]. The area is dominated by well-drained soils with a small component of somewhat poorly drained soils located primarily southwest of the Yakima River in the western portion of the AVA. It has the seventh deepest soils.

Climatic distinction for Yakima Valley AVA was established as warmer than mountains to the west and cooler than areas to the north and east [34]. Heat accumulation does decline precipitously within 24 km to the west of the AVA boundary as elevations climb into the Cascade mountain range (data not shown) and Walla Walla Valley, Red Mountain and Wahluke Slope AVAs, all lying north or east of Yakima Valley, record more GDD than Yakima Valley AVA. Mean FFD was fifth lowest amongst IPNW AVAs potentially due to the flat terrain and associated cold air pooling. Red varieties are dominated by the Bordeaux varieties Merlot and Cabernet Sauvignon and white varieties by the Burgundy variety Chardonnay and Rhine variety Riesling. There are also contingents of the cooler varieties Gewürztraminer and the Burgundy variety Pinot Noir.

Walla Walla Valley AVA, established 7 March 1984 [35], originally consisted of approximately 1,050 km^2^ and was expanded on 27 April 2001 to approximately 1,066 km^2^ [36]. The original appellation was bound by the confluence of the north and south forks of the Walla Walla River, the 610 m contour to its south and east, an approximation of the divide between the Walla Walla and Touchet River drainages to the north and the confluence of the Walla Walla and Columbia Rivers to the west. The expanded appellation includes area in the Touchet River drainage [36], [37].

Distinctive features listed in the Federal Register were limited both for the original and expanded appellation. According to the NED DEM, the range of elevations, 122 to 697 m, within Walla Walla Valley AVA is greater than the stated 250 to 600 m [36]. It also has the greatest proportion of west and northwest-facing slopes, aspects associated with lower heat accumulation and less solar insolation. The loess derived soils were predominately classed as well drained. The AVA has the second highest mean AWC and the highest mean depth to restrictive layer.

The original and expanded petitions both note relatively high precipitation [35], [36]. The AVA has the second highest mean growing season precipitation, just 3 mm fewer than the Columbia Gorge AVA which lays nearly 300 km to the west. PRISM 1971–2000 normals show mean annual precipitation at 44.2 cm and a maximum of 96.1 cm within the AVA (data not shown). This is greater than the mean of 31.8 cm and maximum of 50.8 cm indicated in either final rule description from undisclosed sources [35], [36]. The longest mean growing season is also found here at 200 days, which coincides with the description which states 190 to 220 FFD [35]. However, FFD from PRISM 1971–2000 normals also indicate a minimum growing season of 138 (data not shown). This AVA also shows the second-greatest difference between GDD and BEDD. As expected, the greatest differences between mean GDD and BEDD are found in the warmest AVAs and vice versa. However, the third-warmest AVA, Red Mountain, records only 14C° greater GDD than Walla Walla AVA, but Walla Walla AVA records a 58C° greater difference between GDD and BEDD than Red Mountain AVA. This indicates greater heat accumulation during the peak of the growing season with potentially lower temperatures during budbreak and flowering and after veraison. Walla Walla Valley AVA is mostly planted to red varieties which are dominated by Cabernet Sauvignon with notable area planted with the later-ripening Bordeaux variety Petit Verdot, possibly owing to the long growing season.

The immense Columbia Valley AVA, established 13 December 1984, encompasses approximately 46,000 km^2^ [38]. It is a diverse region and many smaller appellations have been carved from within it since its establishment. Yakima Valley AVA is included within the Columbia Valley AVA, as is Walla Walla Valley AVA following a boundary adjustment [36]. Since many smaller AVAs have been distinguished by more distinctive characteristics, this discussion will not explore Columbia Valley AVA.

Established 11 June 2001, Red Mountain AVA covers approximately 1,650 ha and is contained entirely within Yakima Valley AVA [39]. The Saddle Mountains Formation of the Columbia River Basalt Group underlies the appellation upon which glacial flood sediments have been topped with loess and dune sands [40].

Elevations within Red Mountain AVA range from 167 to 428 m according to the NED DEM, a greater range than the stated 183 to 305 m [39]. Although largely comprised of southwest-facing slopes, the appellation records the fifth lowest mean solar insolation However this is comparable to many other AVAs. This may be partially due to relatively gentle slopes, fourth lowest amongst IPNW AVAs.

Soils of the Warden-Shano association dominate Red Mountain AVA, including Warden silt loam, Hezel loamy fine sand, Scooteney silt loam and Kiona very stony silt loam [39]. Again, this is an area dominated by well drained soils with a component of somewhat excessively drained soils. Soil depths are second only to Walla Walla Valley AVA.

The petitioner stated heat accumulation on Red Mountain AVA exceeds that of other areas in the Yakima Valley [39], a claim confirmed by PRISM GDD. Although Red Mountain AVA is the second-warmest AVA, it has the fifth-least difference between GDD and BEDD indicating more even heat accumulation over the growing season than Walla Walla Valley AVA. The growing season length in the appellation is second only to Walla Walla Valley AVA. Elevation, southwest slopes, continental air mass movement through the gap between Red Mountain and Rattlesnake Ridge to the west and the Yakima River flowing to the west and around the northern boundary of Red Mountain AVA all likely contribute to this long growing season [39]. Like Walla Walla Valley AVA, vineyards are dominated by red varieties, once again Cabernet Sauvignon and notably the Rhône variety Mourvèdre.

Columbia Gorge AVA, like Columbia Valley and Walla Walla Valley AVAs, is the third to share area in both Washington and Oregon. Established 9 July 2004, this roughly 77,500 ha appellation straddles the Columbia River [41]. Slopes are steep in this appellation with the second highest mean. Elevations range from 23 to 839 m with areas well above the 610 m contour defining a portion of the southern boundary [41]. Columbia Gorge AVA records the second lowest mean solar insolation with the greatest standard deviation (data not shown). This is likely due to a greater proportion of north-facing slopes on the Oregon side of the AVA and hillshade effects due to steep slopes.

Columbia Gorge AVA is also dominated by well drained soils and has the fifth lowest mean AWC. This contrasts somewhat with final rule descriptions of slow to moderate permeability and high AWC, although the latter is given no quantitative context [41]. The mean depth coincides well with descriptions from the petitioner [41]. The appellation records the lowest mean pH.

The petitioner states that due to relatively cool conditions, *V. vinifera* grown in the AVA are generally early varietals like Pinot Noir and Gewürztraminer [41]. There is also some area planted to Pinot Gris as well as the generally warmer Burgundy variety Chardonnay. Heat accumulation surfaces place the Columbia Gorge as the coolest IPNW AVA, which also unsurprisingly show the least difference between GDD and BEDD. Growing season precipitation is greatest in this AVA with a large degree of longitudinal variability. Annual precipitation ranges over the strong precipitation gradient agree with the 46 cm on the eastern end and 76 cm on the western end (data not shown) stated by the petitioner [41].

Lying to the south of Yakima Valley and Red Mountain AVAs and north of the Columbia River, the approximately 2,300 km^2^ Horse Heaven Hills AVA was established on 1 August 2005 [42]. It is bound on the west by a 518 m contour and Pine Creek and by the ridge of the Horse Heaven Hills to the north. As seen previously, elevation ranges from the NED DEM (77 to 670 m) are greater than those stated in the final rule description (61 to 549 m) [42], but in this case, the minimum observed elevation in the DEM is greater than the stated minimum. Slopes are comparable to those in Yakima Valley and Red Mountain AVAs, largely face to the south and southeast and solar insolation is second only to the Snake River Valley, which is at a much lower latitude.

Like many IPNW viticultural areas, Horse Heaven Hills soils are composed of aeolian sand and silt, glacial and slackwater sediments, gravel alluvium and Columbia River basalt colluvium, but the proportion and arrangement of these features distinguish appellations from one another [42]. The Horse Heaven Hills are dominated by soils classed as well drained, but also have one of the greatest proportions of somewhat excessively and excessively drained soils. The third deepest soils are also found here.

Ten-year averages of heat accumulation provided in the petition list, from warmest to coolest, Red Mountain, Walla Walla Valley, Horse Heaven Hills and Yakima Valley AVAs [42]. This corresponds to rankings from GDD and BEDD surfaces. Horse Heaven Hills AVA also ranks fourth in difference between GDD and BEDD surfaces again indicating greater heat accumulation at the peak of the growing season. Red varieties planted are again dominated by Cabernet Sauvignon and Merlot with a notable area of the late-ripening Rhône variety Grenache. White varieties include Chardonnay, Riesling, the Bordeaux variety Sauvignon Blanc and the Rhône variety Viognier.

Sitting atop a 24 km long mega alluvial fan deposited by the Missoula Floods, the approximately 33,000 ha Wahluke Slope AVA was established on 6 January 2006 [43]. Noted topographic features are gently south-facing slopes which decline to the east, south and west, providing for good air drainage, while the 451 m contour of the Saddle Mountains provide the northern boundary [43]. Wahluke Slope registers the lowest mean slope, has by far the greatest proportion of south-facing slopes, fifth highest mean solar insolation and the fourth longest growing season. More continuously similar aspects and low slopes reduce hillshade effects and contribute to the levels of solar insolation.

Deep aeolian sands and silts have the fourth deepest soils with relatively large areas of excessively and somewhat excessively drained soils. Drainage classes are split almost evenly between these two classes and well drained soils. These soils also have the lowest AWC and highest pH.

Based on the state of Washington's Public Agricultural Weather System data from 1994–2003, the petitioner states Wahluke Slope AVA is the driest in the region and recorded 1,674 GDD [43]. It does have the lowest mean growing season precipitation and mean annual precipitation of 192 mm based on PRISM 1971–2000 normals (data not shown). The GDD surface indeed rates it as the hottest of all IPNW AVAs, but BEDD only ranks it as the third hottest. Wahluke Slope AVA also shows the greatest difference between GDD and BEDD surfaces. Again, red varieties are dominated by Cabernet Sauvignon and Merlot and white varieties by Chardonnay and Riesling. Other notable varieties are the Bordeaux variety Malbec and surprisingly Gewürztraminer, typically grown in cooler climates.

Fully within Yakima Valley AVA, the nearly 28,000 ha Rattlesnake Hills AVA was established on 20 March 2006. It is comprised of a portion of east-west hills between the Yakima and Moxee River Valleys and lying between the Hanford Reservation and Union Gap [44].

The petitioner states the appellation contains diverse topography along the southern face of the Rattlesnake Hills, much more variability than the rest of the Yakima Valley and most vineyards have been located on southern ridges and terraces with good air drainage [44]. The fourth steepest mean slope and third highest mean solar insolation are found here with greater standard deviations than Yakima Valley AVA (data not shown). Aspects are dominated by south and southwest-facing slopes.

Soils on the Rattlesnake Hills are dominated by those classed as well drained. They contrast with the more coarsely textured sandy soils of Red Mountain and Horse Heaven Hills AVAs, silty soils found elsewhere in the Yakima Valley and are shallow, especially above 335 m where soil formation was above the depositional influence of the Missoula Floods [44]. These finer textured soils have the third highest AWC and are the shallowest of all IPNW AVAs.

The petitioner states that the Umptanum and Yakima Ridges to the northeast shield the Rattlesnake Hills AVA from Arctic fronts, which are funneled toward Red Mountain and Walla Walla Valley AVAs [44]. This contradicts FFD data, which indicates Rattlesnake Hills AVA has the third shortest growing season at a mean of 148 FFD, 28 days shorter than the mean of Red Mountain AVA and 52 shorter than that of Walla Walla Valley AVA. Heat accumulation claims are more accurate; Rattlesnake Hills AVA is substantially cooler than Red Mountain AVA and the area between the two AVAs is cooler than Rattlesnake Hills AVA (data not shown). The appellation also records the second-least difference between GDD and BEDD reflecting both the relatively low heat heat accumulation and indicating even heat accumulation over the growing season. The most common red varieties are again Merlot and Cabernet Sauvignon with a small area of Petit Verdot despite the relatively short growing season. White varieties are largely comprised of Riesling with small areas planted to Viognier and the Loire variety Chenin Blanc.

Straddling the border between Oregon and Idaho and situated in the rift basin that once contained ancient Lake Idaho, the approximately 21,400 km^2^ Snake River Valley AVA was established on 9 April 2007 [45]. Although a 1,040 m contour is stated as its maximum elevation and comprises nearly its entire boundary [45], Snake River Valley AVA contains over 47,000 ha exceeding this elevation according to the NED DEM. A maximum elevation of 1,504 m is located in the western portion of the appellation and many hills and ridges between the 1,040 m contour and drainages exceed the stated maximum elevation. Although the central Snake River Plain is relatively flat as stated in the petition [45], many steeper drainages in northern, western and southern portions of the appellation (data not shown) push mean slope up to sixth steepest. Snake River Valley AVA records the highest solar insolation of IPNW AVAs due to its lower latitude (42°29'N to 45°N). Aspects are fairly evenly distributed in all directions.

The large, edaphically diverse area is also dominated by well drained soils with small components of both somewhat excessively drained and somewhat poorly drained soils. The final rule description provides little in terms of distinctive soil features, but shallow soils are noted and corroborated by SSURGO data with a mean depth greater only than Rattlesnake Hills AVA.

Mean GDD over the AVA were 165C° fewer than the average of four National Climatic Data Center weather station normals from 1971–2000, the same period of record used to compile the GDD surface [45]. Snake River Valley AVA recorded 149 fewer GDD than Walla Walla Valley AVA. While showing less difference than stated by the petitioners assessing AVAs using point measurements, this does generally agree with their claims [45]. The appellation also recorded the second fewest FFD, but exhibits a range second only to the massive Columbia Valley AVA and a maximum of 188 FFD (data not shown). Finding sites with an adequate growing season and heat accumulation are likely the greatest challenges to grape production within Snake River Valley AVA and large areas included in the boundary may never be suitable for *V. vinifera* production.

A third sub-appellation located entirely within Yakima Valley AVA, Snipes Mountain AVA, was established on 20 February 2009. It is the smallest IPNW AVA at less than 1,700 ha. Lying north of the Yakima River, this appellation contains the landform of its namesake as well as 53 ha on Harrison Hill, east of Snipes Mountain, which is stated to have similar soils and contiguous topography with Snipes Mountain [46].

Although Yakima Valley AVA encompasses a much greater elevation range and maximum elevation than Snipes Mountain, including Rattlesnake Hills AVA to the north, Snipes Mountain AVA is substantially higher than its immediate vicinity, particularly to the south, as indicated by the petitioner. Additionally, steep slopes on the southern face of the landforms make Snipes Mountain the third steepest IPNW AVA. However, these steep slopes may lead to shading from adjacent hills. This, in conjunction with gentle northern slopes and the greatest proportion of northern aspects of all IPNW AVAs face, gives Snipes Mountain the third lowest solar insolation.

The appellation is comprised almost entirely of soils classed as well drained. Although the petitioner states that nearly all soils, lacustrine or alluvial, are now generally dry, classified as Aridisols and contain more rock fragments than elsewhere in the Yakima Valley, Snipes Mountain AVA has the highest AWC of all IPNW AVAs. This would be expected to be lower than that of Yakima Valley AVA, which the petitioner states is composed of 43% Mollisols [46].

Climatically, little is stated in the final rule establishing Snipes Mountain AVA aside from steep southern slopes of Snipes Mountain and Harrison Hill draining cold air freely into the Yakima Valley below [46]. It recorded a mean of 6.4 greater FFD than Yakima Valley AVA. It is also relatively warm and dry during the growing season, recording the fourth highest GDD and the second lowest growing season precipitation. Red varieties include Cabernet Sauvignon, Merlot and the Rhone variety Syrah. White varieties again include Chardonnay and Riesling.

The farthest north sub-appellation (47°52'N) within the Columbia Valley AVA flanks the southeastern 19 km of the 88-km-long Lake Chelan, the third deepest lake in the United States from which the appellation is named. Alpine glaciers descended from the Cascade Mountains during the last ice age, 14,000 to 18,000 y.b.p., carving the Lake Chelan Valley, including the lake. Lake Chelan AVA was established on 29 May 2009 and is roughly 9,700 ha [46].

Lake Chelan AVA has the third highest mean elevation of IPNW AVAs and the NED DEM shows greater maximum elevation within the boundary (1,148 m) than the 999 m unnamed peak in the northwest portion of the appellation mentioned by the petitioner [47]. It contains the steepest mean slope with the steepest occurring at higher elevations along the western bank of Lake Chelan and along the northeastern portions of the appellation (data not shown). Along portions of the northern, southern and eastern banks of Lake Chelan, slopes are more gentle as stated by the petitioner [47]. High latitude and steep slopes at the foot of the Cascade Mountains give Lake Chelan AVA the lowest solar insolation.

Distinctions in soil characteristics given by the petitioner focus on differences between the Lake Chelan Valley and the greater Columbia Plateau, primarily greater proportions of volcanic pumice and ash from the eruption of Glacier Peak approximately 12,000 y.b.p. and lower proportions of loess in the top 50 cm of soils. Most notably, soils with large proportions of volcanic ash, pumice or clays weathered from glass have unusually high AWC [47]. This is not corroborated by SSURGO data which ranks Lake Chelan AVA with the second lowest mean AWC. The appellation is dominated by well drained and moderately well drained soils.

Lake Chelan's massive volume and moderating effect are emphasized by the petitioner as defining the appellation's distinctive climate [47]. Despite its high latitude and elevation, the AVA records the fifth highest FFD. Heat accumulation is also moderated by the lake and Lake Chelan is the third coolest IPNW AVA according to GDD and BEDD. As stated by the petitioner, growing season length and heat accumulation drop precipitously as elevations increase to the north and west and, to a lesser extent, to the south and east across the Columbia River and away from the moderating influence of Lake Chelan (data not shown). Red varieties include Pinot Noir, Syrah and the Italian variety Sangiovese. White varieties include Riesling, and Gewürztraminer.

Naches Heights AVA is completely contained by the Columbia Valley AVA and lies to the northwest of Yakima Valley AVA. Bound by the Tieton River to the west, the Naches River to the north and east and Cowiche Creek and the Congdon (Schuler) Canal to the south, the appellation is defined by its elevated andesite plateau. The boundary surrounds approximately 5,300 ha which the petitioner states is the home of 42 ha of grapes [48].

The plateau is largely covered with moderate slopes, but the inclusion of cliffs along the northern, eastern and southern boundaries gives it the eighth highest mean slope. Approaching the Cascade foothills, mean elevation is second only to Snake River Valley AVA. According to the petitioner, the appellation is dominated by Tieton loam and Ritzville silt loam, which are deep soils with adequate drainage [48]. While 98% of the soils in the area are classed as well drained, Naches Heights has the fourth lowest mean depth, which is not excessively shallow at a mean of 142 cm. AWC is fairly high with a mean of 0.169 cm/cm.

Growing season length does decrease to the southwest, across the Tieton River, as claimed by the petitioner [48], but actually increases to the northeast, across the Naches River (data not shown). It is a very cool area with the shortest growing season amongst other IPNW AVAs and second lowest mean GDD and BEDD.

The newest IPNW AVA, Ancient Lakes of Columbia Valley, was established 19 November 2012 and is also completely contained within Columbia Valley AVA. The petition for the establishment of Ancient Lakes AVA utilized GIS data to describe topographic and edaphic features, but relied on individual weather stations for climatic features. As stated, it is a flat appellation with the second-lowest mean slope [49]. Ancient Lakes AVA also has one of the greatest proportion of east-facing slopes. These features may act in opposition in some ways; gentle slopes tend to increase cold air pooling while east-facing slopes are heated earlier in the day while avoiding more intense afternoon sun when temperatures are higher. These factors, a greater elevation and the AVA's higher latitude (46°58'N to 47°16'N) contribute to its moderate heat accumulation compared to nearby Wahluke Slope AVA.

Ancient Lakes AVA ties for second-lowest mean AWC with Lake Chelan AVA. While warmer than Lake Chelan AVA, Ancient Lakes AVA has soil drainage properties similar to those of Wahluke Slope AVA with proportionally 6% and 30% less area classed as somewhat excessively and excessively drained, respectively. It also has the third-lowest growing season precipitation. This indicates a moderate potential for drought stress when comparing these three appellations.

### Principal components analysis

**Figure 3:**
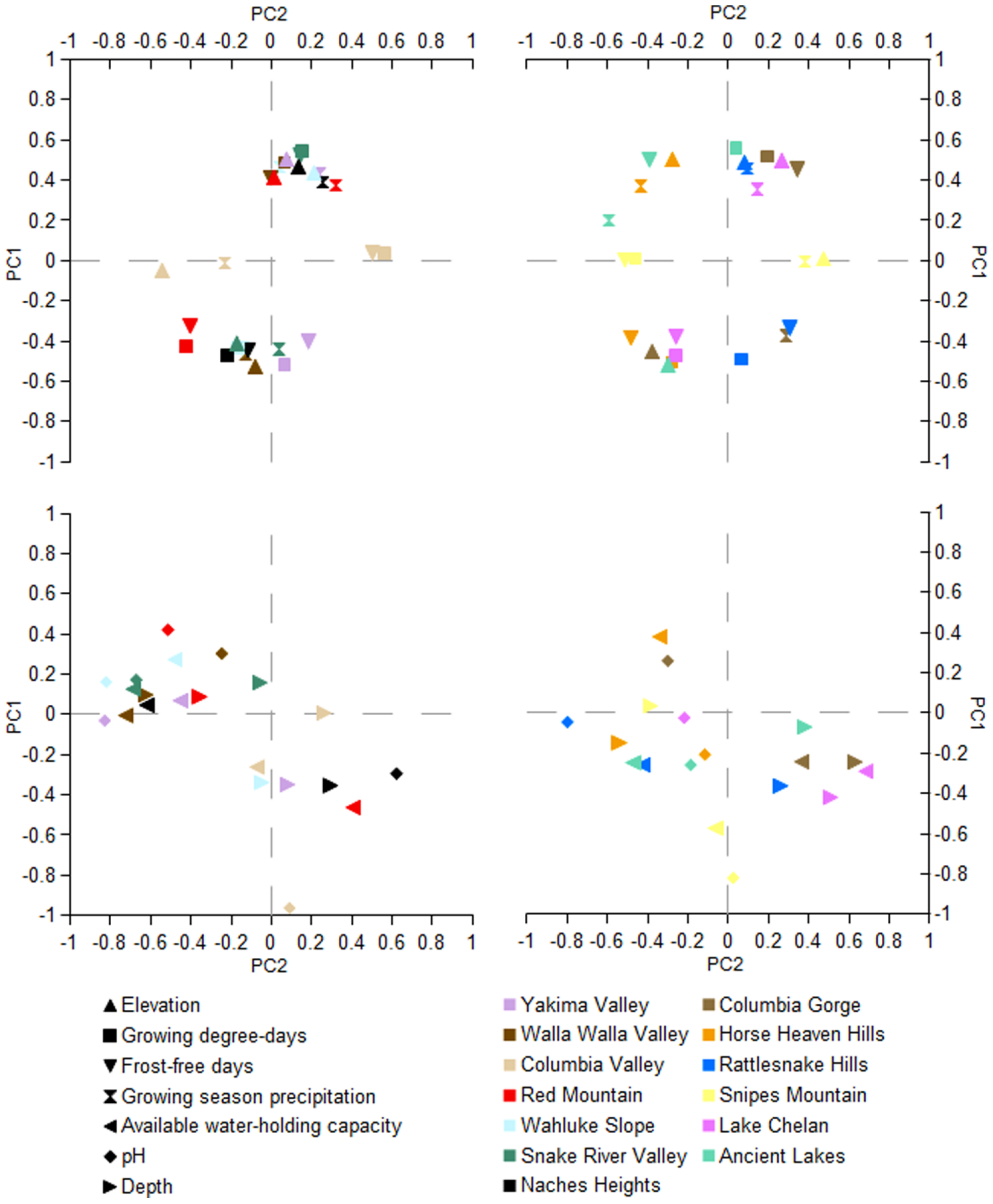
Eigenvectors of first two principal components of seven environmental parameters for 13 inland Pacific Northwest American Viticultural Areas.

Figure 3 shows the PCA results of the first and second principal components of each IPNW AVA with eigenvectors indicating relative loading of each parameter. At least 60% of variance was explained with two principal components and 85% of variance was explained with four principal components for all AVAs. The first principal component for Snipes Mountain AVA explained the greatest amount of variance (80.6%) while the first principal components for Red Mountain AVA and Ancient Lakes AVA explained the least (34.4% and 36.1%, respectively).

A few patterns emerge from this multivariate analysis. With regards to first principal components, Yakima Valley, Horse Heaven Hills, Wahluke Slope, Rattlesnake Hills, Lake Chelan and Naches Heights AVAs showed similar results. These AVAs have large positive eigenvectors for elevation and growing season precipitation and relatively large negative eigenvectors for GDD and FFD. An inverse of this pattern is seen in Walla Walla Valley, Columbia Gorge, Snake River Valley and Ancient Lakes AVAs with relatively large negative eigenvectors for elevation and relatively large positive eigenvectors for GDD and FFD. Walla Walla Valley, Columbia Gorge and Snake River Valley AVAs also show a relatively large negative eigenvector for growing season precipitation, but Ancient Lakes AVA does not. The more moist Columbia Gorge AVA also shows a relatively large positive eigenvector for soil pH. These relationships reflect the interactions between elevation, GDD, FDD and precipitation. While the negative correlation between elevation and both GDD and precipitation are generally well understood, the inclusion of FFD in this interaction is less expected because this parameter tends to rely more on local topography rather than absolute elevations.

The first principal components of Snipes Mountain and Columbia Valley AVAs return the largest eigenvectors for soil parameters. Both have large negative eigenvectors for soil pH and Snipes Mountain AVA also returns a large negative eigenvector for AWC. These two AVAs also returned nearly zero eigenvectors for elevation, GDD, FFD, growing season precipitation and soil depth. The nearly zero eigenvector for precipitation for Snipes Mountain AVA is interesting since it also has an average growing season precipitation of less than 100 mm, but lack of loading implies less influence of this factor than of soil components.

Those AVAs that returned the greatest loadings for elevation, GDD, FFD and growing season precipitation tended to return larger loadings for soil factors in their second principal components and vice versa. Red Mountain, Columbia Gorge, Horse Heaven Hills and Ancient Lakes AVAs. continued to return relatively large eigenvectors for GDD, FFD and PPT. This indicates climatic variables contribute substantially to variance in these appellations.

### Conclusion

Examining existing IPNW AVAs reveals some inaccuracies in their final rulings and quantitative detail in their descriptions not found in their original petitions. Table 4 summarizes key differences found in this study between AVA descriptions in the *Federal Register* and in modeled, geospatial data. Topographic and soil spatial data are easily and freely obtained and should be used more regularly in appellation delineation and characterization. Climatic data is the most difficult to obtain and analyze, but this analysis offers some headway. Indication of data sources when describing climatic characteristics of AVAs, the most temporally variable of distinguishing features, is critical for the relevance of their inclusions. The SSURGO database also includes many other datasets that may be useful in appellation characterization.

**Table 4:** Key differences between American Viticultural Area (AVA) descriptions in Federal Register and observations in modeled geospatial datasets.

Characteristic	Difference(s)	AVA(s) in which difference(s) were observed
Elevation	wider modeled range than stated	Walla Walla Valley, Red Mountain
	higher modeled maximum than stated contour line	Columbia Gorge
	greater modeled maximum than stated	Horse Heaven Hills, Snake River Valley, Lake Chelan
Slope	steeper than stated	Snake River Valley
Frost-free days	modeled minimum much less than stated range	Walla Walla Valley
	far fewer modeled frost-free days compared to Red Mountain and Walla Walla	Rattlesnake Hills
	modeled growing season decreases to the northeast, contrary to description	Naches Heights
Growing degree-days	cooler over the region than mean of four National Climatic Data Center stations	Snake River Valley
Precipitation	greater modeled than stated	Walla Walla Valley
Drainage	well-drained compared to stated 'slow to moderate permeability'	Columbia Gorge

Principal components analyses suggest most AVAs were more strongly influenced by a combination of elevation, GDD, FDD and, in most cases, precipitation. A smaller group showed more influence by soil parameters. However, the PCA did not necessarily coincide with how the AVAs ranked in these components.

Delineation and petitioning for new AVAs in the IPNW and elsewhere should rely more on GIS and spatial datasets in the future. Accurate representation of physical characteristics of AVAs is important in comparisons and understanding of wine regions of the United States. As indicated by the predominance of the most popular varieties grown in the region, planting decisions are likely largely market driven. Often more insight can be gained from less common varieties that are planted and for what wine styles they are intended. Further explanation of vineyard areas by variety and more detailed reporting of varieties planted would help refine descriptions of AVAs. As the IPNW continues to develop its wine grape industry and more distinct AVAs are established, spatially comprehensive descriptions of distinguishing features will help contribute to the understanding of unifying characteristics of the region as a whole as well as the variability within and the breadth of its potential.
